# Anastomotic leak rate and outcome for laparoscopic intra-corporeal stapled anastomosis

**DOI:** 10.4103/0972-9941.62527

**Published:** 2010

**Authors:** Vitali Goriainov, Andrew J Miles

**Affiliations:** Department of Surgery, Royal Hampshire County Hospital, Winchester, Hampshire SO22 5DG, UK

**Keywords:** Anastomotic leak, laparoscopic intra-corporeal anastomosis

## Abstract

**AIMS:**

A prospective clinical audit of all patients undergoing laparoscopic surgery with the intention of primary colonic left-sided intracorporeal stapled anastomosis to identify the rate of anastomotic leaks on an intention to treat basis with or without defunctioning stoma.

**MATERIALS AND METHODS:**

All patients undergoing laparoscopic colorectal surgery resulting in left-sided stapled anastomosis were included with no selection criteria applied. All operations were conducted by the same surgical team and the same preparation and intraoperative methods were used. The factors analyzed for this audit were patient demographics (age and sex), indication for operation, procedure performed, height of anastomosis, leak rate and the outcome, inpatient stay, mortality, rate of defunctioning stomas, and rate of conversion to open procedure. Results for anastomotic leakage were compared with known results from the Wessex Colorectal Audit for open colorectal surgery.

**RESULTS:**

A total of 69 patients (43 females, 26 males; median age 69 years, range 19 - 86 years) underwent colonic procedures with left-sided intracorporeal stapled anastomoses. Of these, 14 patients underwent reversal of Hartmann's, 42 – Anterior Resection, 11 – Sigmoid Colectomy, 2 – Left Hemicolectomy. Excluding reversals of Hartmann's, 29 operations were performed for malignant and 26 for benign disease. Five patients were defunctioned, and 3 were subsequently reversed. The median height of anastomosis was 12 cm, range 4 – 18 cm from anal verge as measured either intra-operatively, or by rigid sigmoidoscopy post-operatively. Four cases were converted to open surgery. There was 1 post-operative death within 30 days. There was 1 anastomotic leak (the patient that died), and 1 patient developed a colo-vesical fistula. Median post-operative stay was 7 days, range 2–19.

**CONCLUSION:**

This clinical audit confirms that the anastomotic leak rate for left-sided colorectal stapled anastomosis is no worse than that for open surgery. Therefore the decision making process for defunctioning stoma should be guided by the same principles as open surgery.

## INTRODUCTION

Following introduction of laparoscopic minimally invasive surgery,[1] the first reports of laparoscopic colectomy appeared in the 1990s.[2–5] However, until recently, laparoscopic colorectal surgery has not been widely accepted and remains available to only a minority of patients. This is partly due to the relatively small number of laparoscopically trained colorectal surgeons and partly due to the lack of evidence documenting the complication rate of the laparoscopic approach in colorectal surgery to convince all colorectal surgeons to adopt it. Colorectal anastomotic leak remains one of the most feared post-operative complications particularly after anterior resection of the rectum with the shift from abdomino-peritoneal resections to total mesorectal excision and primary anastomosis.

Literature states various incidence rates of anastomotic leak in laparoscopic colorectal surgery, ranging from 2.5 to 12%.[6–11] This compares with anastomotic leakage of 1.3-18% following open sigmoid and rectal cancer excision.[8,12–17] We have taken as our standard the anastomotic leak rate in The Wessex Colorectal Cancer Audit for patients in our region of 5.5% for major leak.[18]

Performing proximal defunctioning stoma has been proposed to significantly reduce the effects of anastomotic leakage, if not reduce the anastomotic leak rate itself, in open surgery,[13–15,18–20] but no similar data exist for the laparoscopic colonic surgery. On the other hand, no significant advantage in temporary defunctioning has been demonstrated in open surgery.[9,17,21] Therefore, using a stoma for laparoscopic surgery that would not have been used for open surgery would have negated the anticipated advantages of laparoscopic surgery, including shorter hospital stay, better cosmesis, briefer use of parenteral narcotics and oral analgesics, faster resumption of normal function[22,23] and has been avoided in our practice.

Whilst many investigators have previously assessed laparoscopically assisted extracorporeal stapled anastomoses, our audit reviews intracorporeal anastomoses and is aimed at assessing the safety of laparoscopic anastomosis thereby reducing anxiety regarding the possibility of a higher rate of anastomotic leakage for laparoscopic than open anastomosis.

## MATERIALS AND METHODS

A prospective audit of all patients undergoing laparoscopic surgery with the intention of primary colonic left-sided intracorporeal-stapled anastomosis to identify the anastomotic leak rate on an intention to treat basis.

Between January 2003 and August 2006, all patients undergoing laparoscopic colorectal surgery resulting in left-sided stapled anastomosis were included. All operations were conducted by the same surgical team with the same pre-operative preparation and surgical technique. The factors analyzed for this audit were patient demographics (age and sex), indication for operation, procedure performed, height of anastomosis, leak rate and the outcome, inpatient stay, mortality, rate of defunctioning stomas and rate of conversion to open procedure. Results for anastomotic leakage were compared with known results from the Wessex Colorectal Audit for open colorectal surgery.

Bowel preparation involved clear fluids for 24 h and two phosphate enemas pre-operatively. After mobilization of the left colon, the bowel was tied distal to the tumour with a nylon tape to allow washout of the rectal stump. Division of the colon or rectum was performed distal to the encircling tape using the Ethicon ETS Flex 45 mm or Echelon 60 mm. Firings of these guns were limited to a maximum of two cartridges to whenever possible. An incision was made in the left iliac fossa and the Alexis wound retractor was inserted to facilitate delivery of the bowel and to protect the wound edges from exfoliated malignant cells. The marginal vessels were divided between clips and ligated, a non-crushing bowel clamp applied proximally and the left colon transacted by scalpel. The anvil of the Ethicon 29 mm CDH gun was placed into the end of the bowel and a hand sewn purse string sutured extracorporeally with Prolene 2 ‘O’ suture. The left iliac fossa wound was secured by lifting and clamping the wound retractor and the gun passed per-anum. Anastomosis was performed intracorporeally under direct camera vision with the trocar of the gun being brought through the rectal stump at the site of joining of the two cross staple lines to excise this area of potential weakness. All anastomoses were air tested. An indirect assessment was done by inspecting both the proximal and distal donuts. If there was an air leak or an incomplete donut, a defunctioning ileostomy was used.

## RESULTS

Eighty-four patients (49 females, 35 males; median age 70 years, range 19-89 years) underwent colonic procedures with left-sided intracorporeal-stapled anastomosis. Of these, 15 patients (17.9%) underwent reversal of Hartmann's operation, 54 (64.3%) anterior resection, 11 (13.1%) sigmoid colectomy, 2 (2.4%) left hemicolectomy, 1 (1.2%) total colectomy and 1 (1.2%) reversal of end ileostomy [Table 1]. Thirty-eight operations (56%) were performed to resect bowel cancer and 30 (44%) were for benign disease.

**Table 1:** Number of procedures with known level of anastomosis

0-5 cm	6		Anterior resection
6-10 cm	22	18	Anterior resection
		4	Reversal of Hartmann's
11-15 cm	40	24	Anterior resection
		6	Sigmoid coectomy
		2	Left hemicolectomy
		7	Reversal of Hartmann's
		1	Total colectomy
>15 cm	2	1	Reversal of Hartmann's
		1	Reversal of end ileostomy

The planned laparoscopic procedure with intracorporeal-stapled anastomosis without defunctioning was completed in 73 patients. Sixty-nine patients recovered without significant post-operative complications. One patient was returned to theatre due to an intra-abdominal haemorrhage. One patient suffered a myocardial infarction.

Five cases (6%) were converted to open surgery. Reasons for conversion included small bowel hernia into colostomy site that could not be mobilized, diathermy failure, inability to get stapler across rectum below tumour, excessive adhesions and excessive oozing from dissection field. Seven patients (8.3%) had a primary defunctioning stoma.

**Figure 1:**
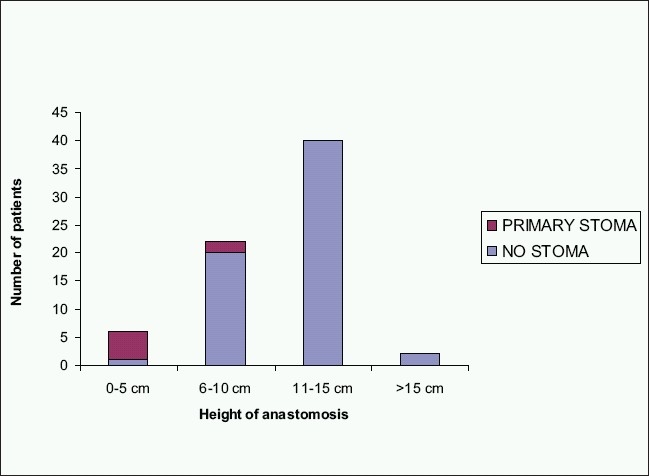
Defunctioning in relation to the level of anastomosis

Intra-operative air leak was evident in one patient, whose anastomosis at 15 cm was oversewn intracorporeally and defunctioned by ileostomy. A subsequent gastrograffin enema x-ray study showed no leakage at 6 weeks post-operatively, and the stoma was closed without complication. In addition, six other patients were defunctioned, and four were subsequently reversed to date. Three patients, two with an anastomosis at 4 cm and one at 6 cm had an incomplete donut and were defunctioned although there was no evidence of air leak on testing. Inspection of these anastomoses by proctoscopy approximately 6 weeks later was satisfactory and the stomas were closed uneventfully without radiological confirmation. One anastomosis was defunctioned as it was judged to be under too much tension despite mobilization of the splenic flexure back to the middle colic artery and one due to too many firings of the cross stapling gun leaving a potential weakness outside the circumference of the TLH donut. One patient was awaiting liver resection for hepatic secondaries and required an uncomplicated recovery from colectomy. Thus, the very low anastomosis at 4 cm was defunctioned to minimize the risk. One patient was converted to open surgery due to excessive generalized ooze compromising laparoscopic vision in the pelvis and was subsequently defunctioned after an open anastomosis. The median height of anastomosis was 12 cm, ranging 4–18 cm from anal verge as measured either intra-operatively or by rigid sigmoidoscopy post-operatively [Figure 1].

Two patients underwent re-operation for complications and had an end stoma formed. An 82-year-old patient with malignant disease whose anastomosis was at 6 cm leaked on the third post-operative day. The patient was returned to theatre within 6 h of becoming unwell; the anastomosis was disconnected and brought out as an end-stoma, but the patient died of overwhelming sepsis within 48 h of re-operation. A 55-year-old patient with an anastomosis at 15 cm developed a colo-vesical fistula following a reversal of Hartmann's operation. The anastomosis was subsequently defunctioned and brought out as an end stoma.

Thirty-day mortality was 50% in the leak group and 0% in the non-leak group.

The median post-operative stay for all 84 patients including those defunctioned, those converted to open surgery and those with complications was 6 days, ranging 2–23.

## DISCUSSION

Since the introduction of laparoscopic surgery, there were concerns about an increased risk of various complication compared to open procedures. In particular, these included potential staging inaccuracies, inadequacy of resection, anastomotic leaks and altered models of tumour dissemination. The technique of fashioning the anastomosis is thought to have considerable influence on the rate of anastomotic failure (hand-sewn vs. stapled: 5.8% vs. 2.3%).[8] In our study, only a stapled technique was used. The height of anastomosis has been shown to affect the incidence of anastomotic leak.[7–9,13,24] In this study, a fatal clinical leak occurred in a relatively low (6 cm) anastomosis. The colo-vesical fistula that developed at 15 cm from the anal margin after reversal of Hartmann's operation is likely to have been due to bladder becoming interpostioned between the anvil and the body of the anastomosis gun during closure rather than failure of an intact anastomosis.

The significance of air testing was stressed by Beard *et al.*[25] In our study, we air tested the anastomoses and inspected the doughnuts peri-operatively. Only one patient had an air leak and this was successfully treated intra-operatively. Whether a defunctioning stoma is necessary in this situation is not known.

While this study did not include radiological assessment of the integrity of anastomoses postoperatively, we believe that only the clinically apparent anastomotic leaks are important. There were only two clinically evident anastomotic leaks post-operatively (2.9%). This compares favourably to the rates of anastomotic leakage of 1.3-17.7% in open procedures elsewhere[8,12–16,26] and 5.5% in our own region.[18] The rate of anastomotic leakage reported from laparoscopic procedures of 2.5-12% previously rarely relates to intra-corporeal anastomosis and usually includes patients with right-sided anastomosis.[6–12,26]

Our results suggest that the anastomotic leak rate for left-sided colorectal stapled anastomosis is no worse than that for open surgery and therefore the decision-making process for defunctioning stoma should be guided by the same principles as in open surgery. Defunctioning is recommended when technical difficulties in completion of the anastomosis are encountered or in the presence of multiple adverse factors.[9,13,27] These include level of anastomosis, gender (males being at higher risk), pre-operative pelvic irradiation, intra-operative adverse events, inadequate bowel preparation or comorbid conditions associated with poor tissue healing.[7,9,13,16,28,27] In our study, the rate of defuctioning was significantly influenced by the level of anastomosis: 83% in 0-5 cm group and 9.1% in 6-10 cm group (*P*<0.01) [Figure 1]. We have used defunctioning stomas in seven patients (8.3%) with no patient who was defunctioned having any clinical evidence of leakage. The one patient that had an anastomotic leak and died might have benefited from a defunctioning stoma although there were no adverse features at the time of surgery to indicate this.

**Figure 2:**
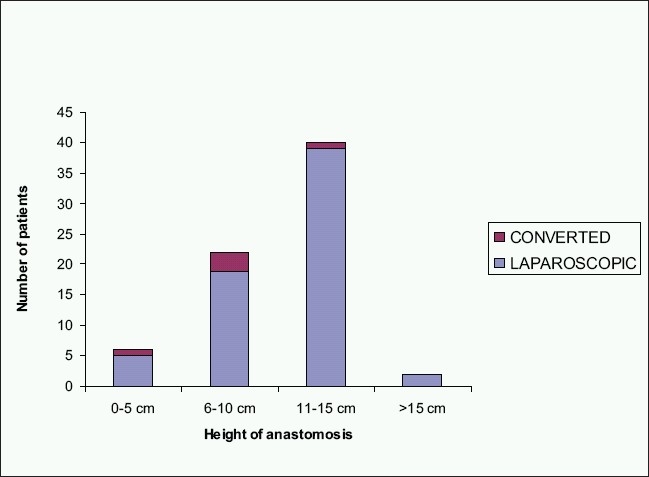
Rate of conversion in relation to the height of anastomosis

The rate of conversion to open surgery has been reported to be between 5.6 and 29%.[5,6,8,12,23,26,29] Although conversion to open surgery should not be considered as a complication, but a prudent choice made to ensure the safety of procedure, our conversion rate of 6% appears at the lower end of expected range. Our results suggest that the conversion rate is not significantly affected by the level of anastomosis [Figure 2].

**Figure 3:**
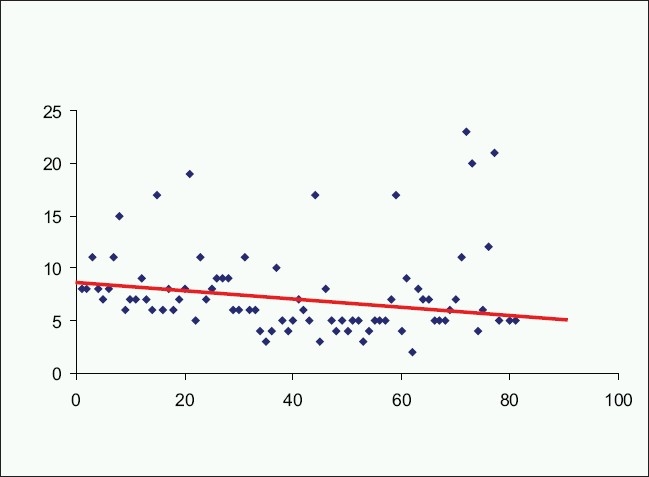
The graph illustrates the general trend (represented by the red line) of post-operative stay becoming shorter as the experience accumulated with increasing number of cases.

In the literature, the length of hospital stay post-laparoscopic/laparoscopically assisted colorectal procedures ranges between 5 and 9 days[6,12,23,26,29] versus post-open procedures 6 and 11 days.[12,23,26,29] The median post-operative hospitalization in our patient group was 6 days (8.5 over the first 2 years, n=28, 5 over the next 2 years, n=53). Thus, our results show statistically significant reduction of inpatient postoperative stay (*P*<0.05) as the confidence and experience of the surgical and nursing teams mature [Figure 3].

In conclusion, we believe that this study demonstrates that the anastomotic leak rate from intra-corporeal laparoscopic anastomosis is no greater than for open surgery or laparoscopic surgery with extra-corporeal anastomosis.
